# Performance and potential utility of the BioFire Joint Infection Panel with synovial fluid and joint tissue specimens

**DOI:** 10.1128/jcm.00594-25

**Published:** 2025-08-26

**Authors:** Shivani Fox-Lewis, Marc Douglass, Sally Roberts

**Affiliations:** 1 Microbiology Department, LabPLUS, Auckland City Hospital 58991, Auckland, New Zealand; Johns Hopkins University, Baltimore, Maryland, USA

**Keywords:** PCR, BioFire Joint Infection Panel, synovial fluid, joint tissue, diagnosis, validation, molecular

## Abstract

The BioFire Joint Infection Panel (BJIP) (bioMérieux) is a rapid sample-to-answer multiplex polymerase chain reaction (PCR) platform for the diagnosis of joint infections. This retrospective study evaluated its performance with synovial fluid and joint tissue specimens from native and prosthetic joints. Joint fluid and tissue specimens received from November 2023 to September 2024 were included. Some specimens were pooled prior to BJIP testing. BJIP results were compared to composite standard laboratory testing (culture, 16S rRNA gene PCR and sequencing [where performed], and concurrent microbiological results for that infection episode). Clinical data were collected (diagnosis, surgical history, antibiotic treatment). Subgroup analysis of native and prosthetic joint specimens was conducted. There were 224 specimens from 134 patients: 107 synovial fluids and 117 joint tissues. The most common organisms, reliably detected by the BJIP, were *Staphylococcus aureus* and *Streptococcus* spp. The on-panel positive percent agreement (PPA) was 90.5% for synovial fluids and 86.8% for joint tissues. There was no significant difference in the PPA for synovial fluids vs joint tissues, nor for native vs prosthetic joints. The additional diagnostic yield was one case. The absence of important pathogens in prosthetic joint infection, e.g., *Cutibacterium acnes*, reduces the overall PPA (74.5% synovial fluid, 77.6% joint tissue). The BJIP performs well in synovial fluid and joint tissue specimens, from native and prosthetic joints for on-panel organisms. There was minimal additional diagnostic yield. We suggest an implementation pathway using the BJIP upfront to provide rapid results to optimize patient care.

**IMPORTANCE:**

This study compared the BioFire Joint Infection polymerase chain reaction panel to standard culture-based methods for detecting organisms causing joint infections. This is a relatively new test, and the data published so far show it performs well with synovial fluid. There are limited published data using it for joint tissue specimens. We found that the BioFire Joint Infection Panel reliably detects pathogens in synovial fluid and joint tissue specimens, though its performance is limited by the absence of important pathogens associated with prosthetic joint infection, e.g., *Cutibacterium acnes*. We suggest using the panel to provide rapid diagnosis to optimize patient care.

## INTRODUCTION

The current gold standard for the microbiological diagnosis of joint infection, as recommended by a number of society guidelines, is bacterial culture, which often fails to identify pathogens due to prior antimicrobial use, infection caused by fastidious organisms, or inadequate sampling techniques (1). Newer guidelines acknowledge the role of molecular diagnostics in providing fast, reliable results that could significantly improve patient care.

The European Bone and Joint Infection Society/Musculoskeletal Infection Society 2021 guideline and the Parvizi criteria 2018 for the diagnosis of periprosthetic joint infection (PJI) include elevated synovial fluid white cell count and synovial fluid and joint tissue culture (2, 3). Molecular testing is not mentioned.

The European Bone and Joint Infection Society 2023 guideline for the diagnosis of septic arthritis of the native joint (SA) includes synovial fluid white cell count and culture, additionally recommending polymerase chain reaction (PCR) testing in culture-negative or antibiotic pre-treated cases (4). Similarly, the British Orthopaedic Association 2024 guideline includes 16S rRNA gene PCR and sequencing (hereafter referred to as 16S) for culture-negative cases (5).

Accurately distinguishing joint infection from non-infectious pathology is crucial for appropriate patient management (1). Though the sensitivity of culture has improved with methods such as implant sonication fluid culture and tissue culture enrichment, the problem of culture-negative cases remains: up to 40% of PJI cases are culture-negative (1, 6).

A meta-analysis reported the sensitivity of culture was 70%–90%, and the sensitivity of PCR for PJI diagnosis was 86% (7). Joint tissue PCR may have a significant role to play in PJI diagnosis, demonstrating higher sensitivity than synovial fluid specimens (95% and 84%, respectively) (7). Intra-operative tissue specimens form a key component of the diagnosis of joint infection; however, the use of PCR with tissue specimens has not been extensively evaluated.

The BioFire Joint Infection Panel (BJIP) (bioMérieux, Marcy-l'Étoile, France) is a rapid multiplexed sample-to-answer PCR platform that detects 29 bacteria, 2 yeast, and 8 antimicrobial resistance gene targets. The BJIP is marketed as having 91.7% sensitivity and 99.8% specificity (8). It has been FDA-approved for use with synovial fluid: published studies show good performance in comparison to culture and 16S (6, 9–11). Importantly, the panel does not detect *Staphylococcus epidermidis* and *Cutibacterium acnes*, common causes of PJI. The BJIP has been shown to have increased diagnostic yield compared with culture (63% vs 26%) (12).

In New Zealand, the most recognized cause of joint infection is *Staphylococcus aureus*. Other common organisms vary between groups (SA vs PJI, adult vs pediatric) but include streptococcus species, coagulase-negative staphylococci, and Gram-negative bacteria (13–16). Data from the Surgical Site Infection Improvement Programme of the Health Quality and Safety Commission New Zealand, for the Te Toka Tumai district, where this study was conducted, show that from 2014 to 2024 hip and knee arthroplasty, the causative organisms for deep surgical site infections were *S. aureus* (0.3%), other staphylococci (0.1%), and non-staphylococcal organisms (0.5%), with a total surgical site infection rate of 1% (17).

PCR may perform better than culture for fastidious organisms, specimens collected while on antibiotic treatment, and in turnaround time to results. This would allow prompt initiation of targeted antimicrobial therapy, or cessation of antimicrobials where infection has been excluded, benefitting individual patients and antimicrobial stewardship efforts (1, 18). A retrospective study estimated that the BJIP would have changed management in 9.7% of cases (19).

This study evaluated the performance of the BJIP with synovial fluid and joint tissue specimens, describing the positive and negative percent agreement and additional diagnostic yield compared with standard laboratory testing.

## MATERIALS AND METHODS

### Specimens

This retrospective observational study was conducted at Auckland City Hospital, a tertiary referral orthopedic service. Table 1 describes the patient demographic included in this study.

**TABLE 1:** Demographic and clinical characteristics of patients included in this study^*a*^

Category	Characteristic	Number (%)
Age (years) (*n* = 134)	<15	17 (13)
	15–30	10 (7)
	30–45	17 (13)
	45–55	15 (11)
	55–65	28 (21)
	65–75	23 (17)
	75–85	22 (16)
	>85	2 (1)
Sex (*n* = 134)	Female	59 (44)
	Male	75 (56)
Ethnicity (*n* = 134)	Asian	16 (12)
	European	83 (62)
	Māori	15 (11)
	Pacific	18 (13)
	Not recorded	2 (1)
Specimen type (*n* = 224)	Synovial fluid	107 (48)
	Joint tissue	117 (52)
Specimen site (*n* = 224)	Knee	120 (54)
	Hip	51 (23)
	Shoulder	16 (7)
	Elbow	11 (5)
	Ankle	6 (3)
	Wrist	9 (4)
	Other	11 (5)
Joint infection diagnosed (*n* = 224)	Yes	112 (50)
	SA	39 (35)
	PJI	73 (65)
	No	112 (50)

^*a*^ Pacific includes Samoan, Tongan, Niuean, Cook Island Maori, Fijian. MELAA is Middle Eastern Latin American and African. Joint infection “No” includes inflammatory diagnoses, non-infection diagnoses, and infections outside the joint space.

Joint tissue pre-processing methods were assessed in our laboratory prior to this study: macerated tissue, macerated and enzymatically digested tissue, and nucleic acid extract from macerated and digested tissue. All three pre-processing methods gave satisfactory results in verification studies. Macerated tissue was used in this study as it was the least labor-intensive pre-processing method. See the supplemental material for our tissue maceration standard operating procedure.

All synovial fluid and joint tissue specimens received from November to December 2023 and March to September 2024 were eligible for inclusion in this study. Inclusion of specimens was via convenience sampling of those that could be retrieved from storage and were of adequate volume.

### Data collection

Specimens were tested using standard laboratory procedures (SLP), including microscopy and culture, with the option of 16S (Sanger sequencing) or referral to another laboratory for targeted PCR if required (see the supplemental material for details of methods included in SLP).

BJIP testing was conducted later to fit with laboratory workflow. SLP testing was not disrupted for this study. Specimens were stored according to laboratory protocols and refrigerated at 4°C until BJIP testing was conducted. BJIP results were not communicated to the clinical teams except at the discretion of the clinical microbiologist. Specimens were tested on the BJIP according to the manufacturer’s instructions, using 0.2 mL of specimen, run on the BioFire FilmArray TORCH system (20).

To maximize the number of specimens that could be included in this study, the following strategy was employed to pool specimens:

- Joint tissue specimens from the same patient, same site, same collection, and same SLP result (all with no organism identified, or all with the same organism and antimicrobial susceptibility profile)
- Synovial fluid specimens from the same patient, same site, same collection, and same SLP result (all with no organism identified, or all with the same organism and antimicrobial susceptibility profile)
- Synovial fluid specimens from different patients with a white cell count <20,000 E+6/L that had negative results (no organism identified), provided there was sufficient volume for repeat individual testing if needed

To form pools, 0.1 mL of each specimen was aliquoted to a maximum of five specimens in a pool. Then 0.2 mL of the pool was used for BJIP testing. If any pool was positive, each individual specimen was retrieved and tested individually by the BJIP. A *post hoc* analysis of the data from this study revealed comparable positive percent agreement for pooled specimens compared to individually tested specimens (see the supplemental material for analysis).

Laboratory data collected included specimen type, date collected, age, sex, date tested, test performed, and result for all SLP tests. BJIP test results, pooling, and date tested were recorded. Clinical data collected from patient records included the joint pathology diagnosis as determined by the treating clinician, antibiotic treatment, prosthetic or native joint, and ethnicity. Infection episodes were determined from the patient record by reviewing dates of admission, surgery, clinical events, and discharge. All data were collected in Microsoft Excel spreadsheets and de-identified prior to analysis.

### Data analysis

BJIP results were compared to composite SLP results. Specimens in this study were not necessarily the first specimen collected during the infection episode and may be culture-negative because of prior antibiotic treatment. To account for this, microbiological results from previous and concurrent specimens during the same infection episode, as well as SLP results for the study specimen, were included, comprising a composite SLP.

A true positive was defined as BJIP detection of the same organism as detected in SLP. The BJIP has genus targets for *Streptococcus* spp. and *Candida* spp., as well as targets for some species within these genera. Where only the genus targets were detected, they were categorized as true positive if the species detected in SLP was not on the BJIP panel. True negative specimens were BJIP-negative with no growth in SLP. Specimens that were BJIP-positive and SLP-negative underwent 16S; if confirmed, they were categorized as true positive, and if unconfirmed, they were categorized as false positive. If 16S had not already been performed as SLP, it was performed for discrepant BJIP results. False negative results were BJIP-negative and SLP-positive. This included growth of off-panel organisms that are not included in the BJIP assay targets (as opposed to on-panel organisms that are among the targets in the assay).

Anaerobic organisms were assessed as one category. These organisms are often grown in polymicrobial culture and are reported as mixed anaerobes. This meant that individual anaerobic target detection by the BJIP could not be consistently compared to SLP results; hence, anaerobic organism detection was analyzed as a group.

Subgroup analysis of SA and PJI specimens was conducted. Examples of infections that were excluded from these categories are osteomyelitis, wound infections, cellulitis, and infected bursitis.

Data collection and analysis were conducted using Microsoft Excel version 2022. Simple descriptive statistics were applied, and statistical analysis was conducted using the chi^2^ test of proportions.

### Operational considerations

Of 107 synovial fluid specimens, a white cell count was recorded for 65 (the remainder were clotted or heavily blood-stained). The white cell count did not correlate with the diagnosis of infection. However, in the absence of an infection diagnosis and crystal arthropathy, the white cell count was <50,000 E+6 cells/L in 95% of specimens.

Specimens were stored refrigerated until BJIP testing occurred, which was up to five months after collection (range 0–159 days, median 24 days), with no impact on the positivity rate (Pearson’s correlation coefficient 0.89 for number of days’ delay in testing and BJIP true positives).

## RESULTS

There were 224 specimens from 134 patients, of which 107 were synovial fluid and 117 were joint tissue specimens. Patient demographics are described in Table 1.

Specimens were mainly from men (56%) aged 55–85 years old (54%) of European ethnicity (62%). The specimens were evenly split between synovial fluid and joint tissue, and 77% were from hip and knee joints. Joint infection was diagnosed for half of the specimens, 65% PJI and 35% SA.

### Performance with synovial fluid and joint tissue

**Fig 1:**
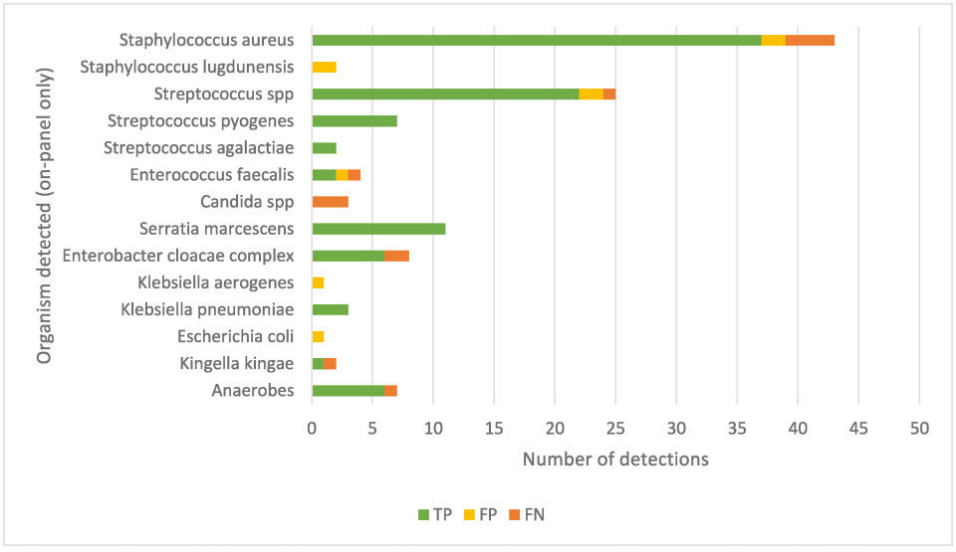
On-panel BJIP organism detection categorized in comparison to the composite SLP. TP is true positive, FP is false positive, and FN is false negative. True negatives are not shown in this figure.

In total, there were 257 BJIP results (greater than the total number of specimens due to multiple organisms detected in some specimens), of which 121 were true negative and 17 were off-panel organisms (false negative). Of the remaining 119 results, 97 (82%) were true positive, and the common organisms detected were *S. aureus* and *Streptococcus* spp. (Fig. 1).

The positive percent agreement (PPA) and negative percent agreement (NPA) of BJIP results with the composite SLP are shown for synovial fluid and joint tissue specimens for on-panel organisms (Table 2). There were 105 BJIP detections of on-panel organisms from synovial fluid and 135 from joint tissue specimens.

**TABLE 2:** Performance characteristics of the BJIP in comparison to composite SLP with synovial fluid and joint tissue specimens for on-panel organisms^*a*^

	Synovial fluid (*n* = 105)		Joint tissue (*n* = 135)		*P*
	Composite SLP positive	Composite SLP negative	Composite SLP positive	Composite SLP negative	
BJIP-positive	38	1	59	8	
BJIP-negative	4	62	9	59	
PPA % (95% CI)	90.5 (81.6–99.4)		86.8 (78.7–94.8)		0.095
NPA % (95% CI)	98.4 (95.3–100)		88.1 (80.3–95.8)		0.161

^*a*^ CI is confidence interval.

With off-panel organisms included, the overall synovial fluid PPA was 74.5% (95% confidence interval 62.5%–86.5%), and the overall joint tissue PPA was 77.6% (95% confidence interval 68.2%–87.0%).

There was no statistically significant difference in the PPA and NPA between synovial fluid and joint tissue specimens (*P* = 0.095 and *P* = 0.161 respectively).

Antimicrobial resistance genes were detected in five of 224 specimens; in all cases, this was *mecA/C* and SCCmec right extremity junction (MREJ). In three of five specimens culture grew methicillin-resistant *S. aureus*; in one specimen the *S. aureus* cultured was penicillin-susceptible; in one specimen no *S. aureus* was cultured.

### Subgroup analysis of SA and PJI

A subgroup analysis of all SA (*n* = 39) and PJI (*n* = 73) specimens was conducted. Table 3 shows the organisms detected and performance characteristics of the BJIP in these subgroups. There were 35 true negative results and nine off-panel organism results.

**TABLE 3:** BJIP organisms detected by SA and PJI subgroups and performance characteristics for on-panel organisms^*a*^

BJIP organism detected	SA			PJI			*P*
	TP	FP	FN	TP	FP	FN	
*Staphylococcus aureus*	14	0	2	11	0	1	
*Streptococcus* spp.	5	0	0	16	0	0	
*Streptococcus pyogenes*	6	0	0	0	0	0	
*Streptococcus agalactiae*	2	0	0	0	0	0	
*Klebsiella aerogenes*	0	1	0	0	0	0	
*Kingella kingae*	1	0	1	0	0	0	
*Candida* spp.	0	0	0	0	0	3	
*Serratia marcescens*	0	0	0	7	0	0	
*Enterobacter cloacae* complex	0	0	0	3	0	0	
*Klebsiella pneumoniae*	0	0	0	3	0	0	
Anaerobes	0	0	0	3	0	1	
PPA % (95% CI)	90.3 (79.9–100)			89.6 (80.9–98.2)			0.220
NPA % (95% CI)	90.0 (71.4–100)			100 (100–100)			0.503

^*a*^ TP is true positive, FP is false positive, and FN is false negative. True negatives are not shown in this table (9 SA and 26 PJI). CI is confidence interval.

With off-panel organisms included, the overall SA PPA was 82.4% (95% confidence interval 69.5%–95.2%) and the overall PJI PPA was 79.6% (95% confidence interval 68.8%–90.4%).

There was no statistically significant difference in the PPA and NPA between SA and PJI specimens (*P* = 0.220 and *P* = 0.503 respectively).

### Discrepant results

A total of 17 BJIP-positive, culture-negative specimens were of sufficient volume to undergo 16S. For six of 17 specimens there was insufficient PCR product amplified, and for two of 17 specimens no bacterial DNA was amplified. In nine of 17 cases, 16S confirmed the BJIP result as true positive. One case was BJIP-positive (*Streptococcus pyogenes*), confirmed by 16S, with no growth in composite SLP. Thus, the additional yield of the BJIP was one.

False negative BJIP results were considered in two groups: off-panel results and other (Tables 4 and 5). The most frequently grown off-panel organism was *C. acnes*, evenly in SA and PJI.

**TABLE 4:** Off-panel organism (false negative) BJIP results for all specimens, and by SA and PJI subgroup^*a*^

Organism	Total	SA	PJI
*Cutibacterium acnes*	8	2	3
*Staphylococcus epidermidis*	2	1	1
*Pseudomonas stutzeri*	2	0	2
*Bacillus* spp.	2	0	0
*Klebsiella oxytoca*	1	0	0
*Micrococcus luteus*	1	0	0
Gram-positive bacillus failed to identify	1	0	0

^*a*^ The total column includes all specimens (including from patients without joint infection) with identification of that organism via SLP.

**TABLE 5:** False negative BJIP results of on-panel organisms with possible explanations

Case	Organism	Specimen type	Potential explanation
1	*Kingella kingae*	Joint tissue	No growth. Detected via referred targeted PCR on a synovial fluid specimen collected the same day.
2	*Staphylococcus aureus* and *Streptococcus* spp.	Synovial fluid	Rheumatological diagnosis. Contamination likely.
3	*Enterobacter cloacae* complex and *Enterococcus faecalis*	Joint tissue (*n* = 3)	Three specimens, same collection. Culture growth: (i) few colonies on *E. cloacae* complex, (ii) one colony of *E. faecalis*, (iii) few colonies of both organisms. Growth in cultures was considered contamination.
4	*Staphylococcus aureus*	Joint tissue	Growth of one colony on day five of incubation. Unclear clinical significance. Patient treated as SA.
5	*Staphylococcus aureus*	Joint tissue	Growth of one colony. Unclear clinical significance. Patient had other causes for post-operative condition but was treated as PJI to cover just in case.
6	*Candida metapsilosis*	Joint tissue (*n* = 3)	Growth of few colonies from each specimen.
7	Anaerobe	Synovial fluid	Moderate growth. BJIP detected from a joint tissue specimen collected 3 days later.
8	*Staphylococcus aureus*	Synovial fluid	Growth from direct culture plates and treated as SA.

False negative results of on-panel organisms occurred in 12 specimens from eight patients (Table 5). The potential explanations include information from SLP reporting and the patient record regarding how the SLP results were interpreted by treating clinicians.

Case 1 did not grow, and the specimen on which *Kingella kingae* was detected was not available for BJIP testing. Cases 2 to 5 were likely growth of contaminants. Though patients 4 and 5 were treated for joint infection, the significance of the growth in culture was questioned. Cases 6 to 8 have no clear reason apparent for the missed BJIP detection, though the sensitivity of the BJIP varies across its range of targets, including some anaerobes and the *Candida* spp. target. For case 8, a handling error in the laboratory cannot be excluded.

Six specimens had false positive BJIP detections; four were polymicrobial cultures reported as mixed growth including specific organisms, in which true positives were also detected. One patient with gout and no growth in SLP had *S. aureus* detected by the BJIP. One patient had SA with *Streptococcus* spp. true positive and *Klebsiella aerogenes* false-positive detections.

## DISCUSSION

This study compared the BJIP with composite SLP for synovial fluid (PPA 90.5% for on-panel organisms and 74.5% overall) and joint tissue (PPA 86.8% for on-panel organisms and 77.6% overall). The NPA was 98.4% for synovial fluid and 88.1% for joint tissue. The performance of the BJIP was comparable in synovial fluid and joint tissue specimens, with no statistically significant difference found in positive and negative percent agreement between these two groups. Similarly, the BJIP performed equally well in SA and PJI specimens, with no statistically significant difference found.

The performance characteristics of the BJIP found in this study are similar to previous studies for synovial fluid, with on-panel PPA/sensitivity reported around 91% and specificity close to 100% (6, 9, 21). A multicenter study of 1,544 synovial fluid specimens found that most detections were of *S. aureus* and *Streptococcus* spp., as in this study, and reported comparable on-panel sensitivity (90.9%) and specificity (98.5%) (21).

The overall PPA including off-panel organisms found in this study is higher than previous studies, reporting 56%–69% in synovial fluid, indicating that off-panel organisms did not form as big a contribution to our data set compared with other studies (6, 22). Interestingly, there were roughly equal numbers of off-panel results in the SA and PJI groups, of which approximately half in each group were deemed clinically significant by the treating clinicians.

Our study found no significant difference in the PPA between SA and PJI specimens. In contrast, Schoenmakers et al. reported the best sensitivity in SA (83%), then late acute PJI (73%) and worst in early acute PJI (30%) (11). Similarly, Gardete-Hartmann et al. found the clinical sensitivity of the BJIP to be highest for late acute PJI (73%) compared with early PJI (46.6%) and chronic PJI (40%) (19). The PIANO study demonstrated that the epidemiology of PJI differs in these stages, with β-hemolytic streptococci most prevalent in late acute PJI compared with early or chronic, which may contribute to the increased clinical sensitivity of the panel in this group (16).

Prior literature examining the use of multiplex PCR with joint tissue specimens is limited. Unyvero (Curetis AG, Holzgerlingen, Germany) assays have shown variable results, with sensitivity of 30% and 86% in two studies (23, 24). Hoffman et al. reported the sensitivity of the BJIP with joint tissue was 55%, though whether this is overall or for on-panel organisms only is not stated (25). The PPA of the BJIP with joint tissue found in our study is higher than previously reported. The pre-processing of tissue specimens is not detailed in the discussed published studies, and there may be differences that contribute to the increased sensitivity reported here.

Prior studies have reported significant increased diagnostic yield with use of the BJIP (15%–20%) with the detection of fastidious organisms contributing to this (9, 10, 12, 22). In contrast, our study found one additional case diagnosed by the BJIP, giving an additional yield of 1.1%. This likely reflects the epidemiology of SA and PJI in our setting, that almost all cases are caused by staphylococci and streptococci, which are readily isolated in culture.

The authors suggest that for settings with similar epidemiology to ours, the strength of the BJIP is its rapid turnaround time; thus, it is best utilized in cases that are most likely to be culture-positive, used upfront on specimen receipt. Prior studies vary in their recommendations, with Azad et al. supporting the use of the BJIP later in the diagnostic pathway, once cultures have been shown to be negative and no other microbiological evidence of a pathogen, but Berinson et al. supporting upfront use of the BJIP (6, 26). The BJIP must be used as an adjunct to SLP, since culture is still necessary for off-panel organism growth, phenotypic susceptibility testing, and typing.

*C. acnes* was the most common off-panel organism in this study. The absence of key off-panel organisms, particularly coagulase-negative staphylococci, is a known limitation of the BJIP and, as seen in this study, mostly impacts the diagnosis of PJI. The on-panel false negatives are mainly explained by growth of probable contaminants. The BJIP Instructions for Use report low sensitivity for the *E. cloacae* complex, *Parvimonas micra,* and *Candida* spp. targets, which occurred as false negatives in this study. The *Candida* spp. target is expected to detect *C. metapsilosis* cultured in this study.

The most common organisms detected were *S. aureus, S. pyogenes,* and other *Streptococcus* spp., as expected from prior studies in New Zealand (13–16). Gram-negative organisms such as *Serratia marcescens, E. cloacae* complex, *Klebsiella* spp., and anaerobes were present in PJI specimens and not in SA. There were two cases of *K. kingae* in this study, of which one was detected by the BJIP. The other was culture-negative and detected by targeted PCR at another laboratory. In our region, we expect approximately one to two *K. kingae* culture-positive specimens per year [Internal laboratory database search, 27/11/2024].

Antimicrobial resistance genes were only detected in five of 224 (2%) specimens. Most of these detections correlated with methicillin-resistant *S. aureus* growth in culture. One specimen cultured a penicillin-susceptible *S. aureus*, and the BJIP detection here likely represents an empty cassette. One specimen did not culture *S. aureus*, though a swab from the same site and day was reported as growth of mixed Gram-positive and Gram-negative bacteria; thus, it is possible that *S. aureus* may have been present as part of mixed growth. Though not demonstrated in this study, the BJIP antimicrobial resistance gene targets may play a role in patient treatment and surveillance of antimicrobial resistance.

Strengths of this study include the good sample size, the evaluation of joint tissue specimens as well as synovial fluid, and the inclusion of all specimens received for the diagnosis of infection to try to maximize the chances of detecting the potential additional yield of the BJIP. This study correlated clinical data with laboratory results, informing on the joint pathology diagnosis and clinical management.

Limitations of this study include that it is a single-center retrospective study. Specimens were included by convenience sampling. Clinical data collection was from the patient record which may affect the completeness and accuracy of the data. The subgroup analysis of SA and PJI specimens shows good performance characteristics, though the sample sizes in these subgroups are small as reflected in the wide confidence intervals. Pooling of specimens did not result in a lower PPA (data in the supplemental material); however, a definitive comparison of pooling versus individual testing is outside the scope of this study.

At our laboratory, cultures from synovial fluid and joint tissue specimens are incubated for 7 days before the final report is issued. The BJIP could offer significant gains in time to reporting organism identification and common antimicrobial resistance genes, supporting prompt diagnosis for optimal surgical and antimicrobial management (18, 26). Sangaletti et al. propose incorporating the BJIP into a surgical decision algorithm for suspected PJI (27).

The BJIP is a rapid sample-to-answer platform capable of diagnosing many common causes of SA and PJI. This study shows that it performs reliably in synovial fluid and joint tissue specimens. With appropriate diagnostic stewardship, the BJIP could be a valuable asset to clinical laboratories supporting the diagnosis of joint infections. Future studies are needed to determine its impact on surgical and antimicrobial management and stewardship, as well as its health economic impact.

## SUPPLEMENTAL MATERIAL

Supplemental Material jcm.00594-25-s0001.docx Detailed SLP, maceration SOP, and pooling analysis.

ASM does not own the copyrights to Supplemental Material that may be linked to, or accessed through, an article. The authors have granted ASM a non-exclusive, world-wide license to publish the Supplemental Material files. Please contact the corresponding author directly for reuse.
