# Supplementary material for: Performance and potential utility of the BioFire Joint Infection Panel with synovial fluid and joint tissue specimens
Source: J Clin Microbiol. 2025 Aug 26;63(10):e00594-25. doi: 10.1128/jcm.00594-25 (PMC12505999; doi:10.1128/jcm.00594-25)
Supplement: Supplemental Material — Detailed SLP, maceration SOP, and pooling analysis. [file jcm.00594-25-s0001.docx]

**Tissue maceration procedure**

Tissue maceration is conducted using sterile equipment and the Dispomix tissue homogeniser.

1. In the biological safety cabinet, with sterile forceps, remove the tissue to a sterile petri dish. Dissect the specimen and choose an areas with visible pus and take a representative portion for culture; aim for 0.2-4g, usually 1g will be sufficient.
2. Macerate the pieces of tissue and place into the sterile mixer tube.
3. Add an appropriate amount of sterile saline (0.3-2ml) to assist with maceration.
4. Check the correct profile is displayed on the Dispomix (use Routine, profile 9) and press the green start button.
5. After homogenisation is complete remove the tube from the Dispomix and transfer to the biological safety cabinet.
6. Unscrew the lid to remove the homogenate directly. At no point should the operator attempt to remove the homogenate by piercing the sterile rubber septum with a pipette. This area of the tube will not be sterile.
7. Inoculate one drop of homogenised tissue material onto the required culture media.

**Standard laboratory procedures**

Standard laboratory procedures (SLP) for the diagnosis of joint infections involves the following for each specimen type:

- Synovial fluid:
  - Cell count (1:10 dilution for cell count), with differential if the white blood cell count is ≥ 2000 E+6/L
  - Gram stain
  - Microscopy for crystals
  - Culture (sheep blood agar in CO_2_, GC Saponin agar in CO_2_, anaerobic culture in brain heart infusion agar, inoculation into a BD BACTEC Peds Plus/F bottle)
- Joint tissue – macerated (see procedure above) and homogenised in saline for:
- Gram stain
- Culture (sheep blood agar in CO_2_, GC Saponin agar in CO_2_, MacConkey agar in O_2_ and anaerobic culture in brain heart infusion agar, also supplemented with kanamycin-vancomycin and nalidixic acid)

All samples (synovial fluid or tissue, native or prosthetic joint) are incubated for seven days.

If samples are culture-negative they may undergo either or both of the following, at the discretion of the clinical microbiologist:

- PCR testing at another laboratory. These specimens are tested on a number of syndromic multiplexed commercial panels: AusDiagnostics (Sydney, Australia) panels or the Xpert Xpress Strep A (Cepheid, USA) for *Streptococcus pyogenes.* Possible organisms that could be tested for by targeted PCR via referral to another laboratory include Kingella kingae, Streptococcus pneumoniae, Staphylococcus aureus, Neisseria gonorrhoeae and Streptococcus agalactiae.
- 16S rRNA gene PCR and sequencing at our laboratory. This is performed via an in-house Sanger sequencing assay.

**Pooling analysis**

In total 95 specimens resulting in 98 organism results were tested via pools, and 129 specimens resulting in 159 organism results were tested individually.

Table 1 details the performance characteristics for specimens tested in pools (32 organism results in synovial fluid and 64 organism results in joint tissue) for on-panel organisms and all organisms (on-panel and off-panel). There were two off-panel organism results for specimens tested in pools (both occurred in joint tissue specimens).

Table 2 shows parallel data for specimens tested individually, not in pools, for which there were 73 on-panel organism results for synovial fluid (82 organism results when off-panel organisms are included) and 71 on-panel organism results for joint tissue (77 when off-panel organisms are included).

Table S1. Performance characteristics of the BJIP in comparison to composite SLP with synovial fluid and joint tissue specimens, for specimens tested in pools

|  | **Synovial fluid (n=32)** | | **Joint tissue (n=64)** | |
| --- | --- | --- | --- | --- |
| On-panel analysis only | | | | |
|  | **Composite SLP positive** | **Composite SLP negative** | **Composite SLP positive** | **Composite SLP negative** |
| **BJIP positive** | 9 | 0 | 25 | 0 |
| **BJIP negative** | 0 | 23 | 3 | 36 |
| PPA % | 100 | | 89.3 | |
| NPA % | 100 | | 100 | |
|  | **Synovial fluid (n=32)** | | **Joint tissue (n=66)** | |
| All analysis (on-panel and off-panel) | | | | |
|  | **Composite SLP positive** | **Composite SLP negative** | **Composite SLP positive** | **Composite SLP negative** |
| **BJIP positive** | 9 | 0 | 25 | 0 |
| **BJIP negative** | 0 | 23 | 5 | 36 |
| PPA % | 100 | | 83.3 | |
| NPA % | 100 | | 100 | |

Table S2. Performance characteristics of the BJIP in comparison to composite SLP with synovial fluid and joint tissue specimens, for specimens tested individually (not pooled)

|  | **Synovial fluid (n=73)** | | **Joint tissue (n=71)** | |
| --- | --- | --- | --- | --- |
| On-panel analysis only | | | | |
|  | **Composite SLP positive** | **Composite SLP negative** | **Composite SLP positive** | **Composite SLP negative** |
| **BJIP positive** | 29 | 1 | 34 | 8 |
| **BJIP negative** | 4 | 39 | 6 | 23 |
| PPA % | 87.9 | | 85 | |
| NPA % | 97.5 | | 74.2 | |
|  | **Synovial fluid (n=82)** | | **Joint tissue (n=77)** | |
| All analysis (on-panel and off-panel) | | | | |
|  | **Composite SLP positive** | **Composite SLP negative** | **Composite SLP positive** | **Composite SLP negative** |
| **BJIP positive** | 29 | 1 | 34 | 8 |
| **BJIP negative** | 13 | 39 | 12 | 23 |
| PPA % | 69.1 | | 73.9 | |
| NPA % | 97.5 | | 74.2 | |

The PPA for pooled and individually tested specimens were in agreement, for synovial fluid and joint tissue specimens. Cautious interpretation is advised since the numbers in each subgroup analysis are small. A non-random pooling strategy was utilised, though this should have increased the positive percent agreement in individually tested specimens, which included specimens not pooled because they were expected to be positive. Overall, the data demonstrate that pooling specimens did not produce a large impact on the observed positive and negative percent agreement for each specimen type.
